# Two novel mutations in the *ALPL* gene of unrelated Chinese children with Hypophosphatasia: case reports and literature review

**DOI:** 10.1186/s12887-019-1800-4

**Published:** 2019-11-25

**Authors:** Xiaojian Mao, Sichi Liu, Yunting Lin, Zhen Chen, Yongxian Shao, Qiaoli Yu, Haiying Liu, Zhikun Lu, Huiyin Sheng, Xinshuo Lu, Yonglan Huang, Li Liu, Chunhua Zeng

**Affiliations:** 1 Department of Genetics and Endocrinology, Guangzhou Women and Children’s Medical Center, Guangzhou Medical University, 9 Jinsui Rd, Guangzhou, 510623 China; 2 Department of Radiology, Guangzhou Women and Children’s Medical Center, Guangzhou Medical University, 9 Jinsui Rd., Guangzhou, 510623 China; 3 Department of Dentistry, Guangzhou Women and Children’s Medical Center, Guangzhou Medical University, 9 Jinsui Rd., Guangzhou, 510623 China; 4 Clinical Laboratory, Guangzhou Women and Children’s Medical Center, Guangzhou Women and Children’s Medical Center, Guangzhou Medical University, 9 Jinsui Rd., Guangzhou, 510623 China

**Keywords:** Hypophosphatasia, Alkaline phosphatase, Mutation, Hypomineralization

## Abstract

**Objective:**

Hypophosphatasia (HPP) is an inherited disorder of defective skeletal mineralization caused by mutations in the *ALPL g*ene that encodes the Tissue Non-specific Alkaline Phosphatase (TNSALP). It is subdivided into six forms depending on the age of onset: perinatal lethal, prenatal benign, infantile, childhood, adult, and odonto HPP. Among these, infantile HPP is characterized by early onset and high frequency of lethal outcome. Few studies have reported the phenotype and genetic characteristics of HPP in Chinese children.

**Case presentation:**

Three forms of HPP were identified in four unrelated patients from four different Chinese families, including one lethal infantile (patient 1), two childhood (patient 2 and 3) and one odonto HPP (patient 4). Six variants in the *ALPL* gene were identified, including five missense mutations and one frameshift mutation. Of which, none were reported previously in the Chinese population, and two were novel (c.359G > C: p.G120A and c.1017dupG: p.H340AfsX3). Patient 1 carrying a novel homozygous (c.359G > C) mutation showed respiratory distress and pneumonia at first day of his life. He presented nearly negligible level of serum ALP activity, overall skeletal hypominaralization and died at 3 months old. Patient 2, 3 and 4 were compound heterozygotes with decreased serum ALP activity. Patient 2 and 3 presented premature loss of deciduous teeth, muscle weakness and bone pain, whereas patient 4 had early loss of deciduous teeth only. All four pedigrees exhibited autosomal recessive pattern of inheritance.

**Conclusions:**

In this study, six mutations in the *ALPL* gene were found in four Chinese HPP patients, two of which were novel: c.359G > C in exon 5 and c.1017dupG in exon 10. Our results strongly indicated that the novel mutation c.359G > C might be disease-causing and associated with severe infantile form of HPP.

## Background

Hypophosphatasia (HPP) is a rare hereditary disorder caused by loss of function mutations in the *ALPL* gene that encodes the Tissue Nonspecific Alkaline Phosphatase (TNSALP), one of the alkaline phosphatase (ALP) family members [1–3]. TNSALP is predominantly expressed in the liver, skeleton, kidney and teeth [4]. Its specific function is to cleave the extracellular substrates including inorganic pyrophosphate (PPi), pyridoxal-5-phosphate (PLP) and phosphoethanolamine (PEA) [5]. The deficiency of TNSALP causes the extracellular accumulation of PPi, a potent inhibitor of mineralization, resulting in the defective teeth and bones.

HPP is clinically characterized by decreased level of serum ALP activity and defective skeletal mineralization. It is subdivided into six forms depending on the age at diagnosis: perinatal lethal, prenatal benign, infantile, childhood, adult, and odonto HPP [6]. The patients with lethal perinatal form show markedly impaired mineralization in utero, whereas the patients with infantile form mostly present respiratory complications, widespread demineralization and rachitic symptoms during the first 6 months of life, both of which are defined as severe forms of HPP [6]. The childhood form of HPP displays milder symptoms between 6 months and 18 years with premature loss of primary teeth, delayed walking, short stature and bone deformities. As one of the mildest forms of HPP, odonto HPP is characterized by premature exfoliation of primary and/or severe dental caries without abnormalities of the skeletal system.

Both autosomal dominant and recessive transmission have been shown in HPP. In general, the more severe the HPP is, the more often it could be recessive inheritance. For instance, lethal perinatal form and most infantile forms of HPP are recessively inherited, whereas less severe forms, including perinatal benign, childhood, odonto and adult forms of HPP, show both dominant and recessive inheritance [7]. It has been reported that severe HPP has lower prevalence (1/300,000) than that of less severe forms of HPP (1/6370) [8]. Further, more severe forms of the HPP present lower serum AP activity levels. The studies all indicated that the severity of the HPP is correlated with the activity level of ALP, which is encoded by the *ALPL* gene [9].

The diagnosis of HPP is based on low level of serum ALP activity and genetic testing of the *ALPL* gene mutations. To date, a total of 390 disease-causing mutations in the *ALPL* gene have been reported, most of which are missense mutations (70.3%) (http://www.sesep.uvsq.fr/03_hypo_mutations.php). It has been suggested that large variety of mutations results in variable deficiency of ALP activity and distinct clinical phenotypes [6, 10]. Although genotype-phenotype correlation has been observed, it needs more clinical and genetic data to support. Particularly, there are few pediatric HPP patients reported in the Chinese population.

In the present study, we characterized the distinct clinical and mutational features of four unrelated Chinese children with different forms of HPP, and explored the correlations between the phenotype and genotype in a lethal infantile HPP with a novel homozygous *ALPL* mutation.

## Case presentation

### Clinical features

**Fig. 1:**
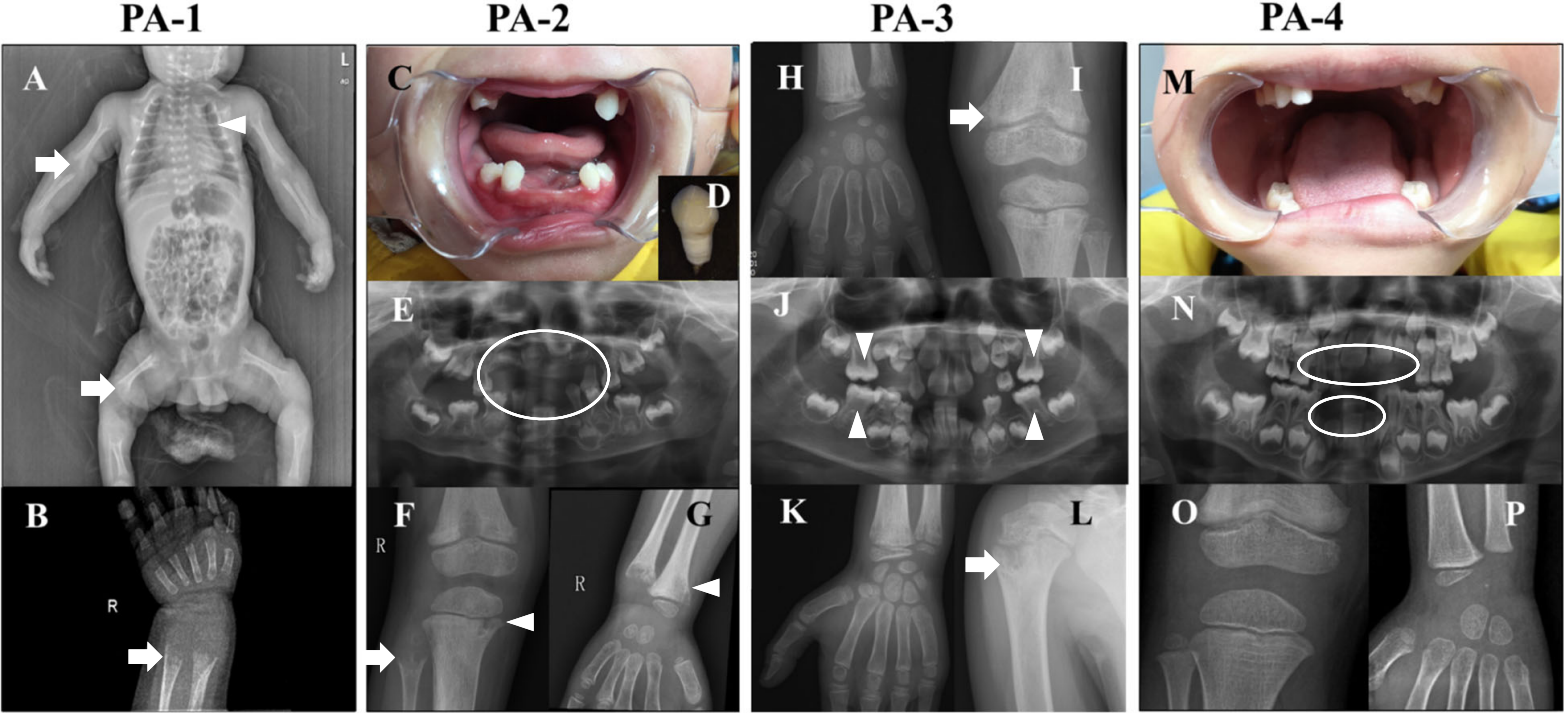
Photos and X-rays of teeth and bones in 4 HPP children with HPP. **a** & **b** X-rays show general hypomineralization, gracile ribs (arrowhead), small clavicles, shortened long bones and flared metaphysis (arrow) in patient 1 (PA-1) at 2-month-old; **c**, **d** & **e** Premature loss of deciduous tooth (circle) in Patient 2 (PA-2) at 2-year-old; **f** & **g** X-rays show flared metaphysis (arrow), and bone destruction (arrowhead) in PA-2; **h** & **i** X-ray shows hypomineralization (arrow) in PA-3 at 4-year-old; **j** Panoramic X-ray in PA-3 at 8-year-old shows decreased mineralization of the alveolar bone, enlarged pulp chambers (arrowhead) and alveolar bone resorption; **k** & **l** Bone destruction in the proximal humerus (arrow) of PA-3 at 8-year-old; **m** & **n** Premature loss of deciduous tooth (circle) in Patient 4 (PA-4) at 4-year-old; **o** & **p** X-ray didn’t show significant bone abnormality

The clinical phenotypes of all four patients with HPP (three males and one female) from four unrelated families are summarized in Table 1 and Fig. 1. They were all born to nonconsanguineous parents. They were initially referred to our clinic due to variable clinical manifestations, including growth failure, premature loss of teeth and rachitic symptoms. With biochemical tests, all patients showed remarkably decreased levels of ALP activity (Table 1). All patients were then suspected and finally diagnosed as HPP with confirmation of disease-causing mutations in the *ALPL* gene.

**Table 1:** Clinical features of 4 HPP patients at diagnosis

Patient	PA-1	PA-2	PA-3	PA-4
Gender	M	F	M	M
Age of onset	1d	1 y	1y	1.5y
Age at diagnosis	2m	2.4y	8y	4y
Form of HPP	Infantile	Childhood	Childhood	Odonto
Serum ALP (U/l) (reference 118-390)	5	42	67	42
Calcium (mmol/L) (reference 2.2-2.7)	3.1	2.4	2.5	2.2
Serum Phosphate (mmol/L) (reference 1.3-1.9)	2.1	2	1.7	1.66
Serum PTH (pmol/L) (reference 1.2-7.1)	0.32	0.32	1.56	0.36
25-hydroxyvitamin D(nmol/L) (reference 50-150)	na	78.2	54.4	71
Height (SD)	<-2SD	0SD	0~-1SD	0~-1SD
Failure to thrive	Y	N	N	N
Waddling gait	NA	Y	Y	N
Joint pain	N	N	Y	N
Early loss of deciduous teeth	NA	Y	Y	Y
Hypomineralisation	Y	Y	Y	Y
Loss of bone	Y	Y	Y	N
Deformity of long bones	Y	N	N	N
Flared metaphyses	Y	Y	Y	N
Nephrocalcinosis	N	N	N	N
Enzyme replacement	N	N	N	N
Outcome	Dead	Alive	Alive	Alive

Patient 1 (PA-1) was delivered at term by c-section with a birth weight of 3000 g. He experienced respiratory distress and newborn pneumonia soon after he was born. He presented with feeding difficulty, seizure, poor head control and failure to thrive during the first 2 months of his life. He showed underweight, short stature, enlargement of the anterior fontanelle, and developmental delay when he was diagnosed at 2 months old. His serum ALP activity was almost undetectable (Table 1). X-ray showed general hypomineralization, deformity of long bones, flared metaphyses and hypolucent mid-metaphyses (Fig. 1a and b). He died of pneumonia and respiratory failure at 3 months old. Patient 1 was classified as infantile HPP (Table 1).

Patient 2 (PA-2) began to present premature loss of the deciduous teeth and muscle weakness at 1 year old. She had waddling gait and delay of walking. She had lost most of her deciduous teeth and only reserved seven teeth when she came to the clinic at 2 years old (Fig. 1c and d). Very low level of serum ALP activity (42 U/L) suggested the diagnosis of HPP (Table 1). Panoramic radiographs showed premature loss of most of deciduous teeth, taurodontism in the deciduous molars and maldevelopment of permanent teeth (Fig. 1e). X-ray of wrist and knee revealed flared metaphyses and bone destruction in distal femur and proximal tibia (Fig. 1f and g). Patient 2 was diagnosed as childhood HPP at 2 years and 5 months old (Table 1).

Patient 3 (PA-3) began to present joint swelling, bone pain and muscle weakness at 1 year old. He experienced delay of walking and early loss of deciduous teeth. His symptoms of joint swelling and bone pain were not relieved at 8 years old. He had body weight and height within normal ranges. Serum ALP activity was 41 U/L at 4 years old and 67 U/L at 8 years old. Panoramic X-ray showed taurodontism, reduced alveolar bone, and enlarged pulp chambers and root canals (Fig. 1j). The X-ray revealed hypomineralization at 4 years old (Fig. 1h, i) and bone destruction in the proximal humerus at 8 years old (Fig. 1k, l). Patient 3 was diagnosed as childhood HPP at 8 years old (Table 1).

Patient 4 (PA-4) experienced premature loss of deciduous teeth since he was one and half years old. The physical examination was unremarkable, other than the absence of the upper and lower anterior incisor and canine teeth (Fig. 1m). Serum ALP activity was 42 U/L at diagnosis when he was 4 years old. Panoramic X-ray showed a reduced alveolar bone of whole dentition (Fig. 1n). His wrist and knee X-ray revealed normal growth plates without any evidence of defective bones (Fig. 1o and p). Patient 4 was diagnosed as odonto HPP at 4 years old (Table 1).

### Mutational analysis

In order to confirm the diagnosis of HPP and correlate the phenotype with specific genotype, mutational analyses of the *ALPL* gene were performed in all four patients and their parents except the mother of patient 1 was not involved in this study. The *ALPL* variants in patient 1 and patient 3 were identified using next generation sequencing and confirmed with Sanger sequencing, whereas the variants in patient 2 and patient 4 were identified and confirmed by direct Sanger sequencing. A 3D structural modeling of the TNSALP constructed based on its sequence homology to the placental isozyme (PDB ID: 1EW2), was used to locate the missense mutation [11].

**Fig. 2:**
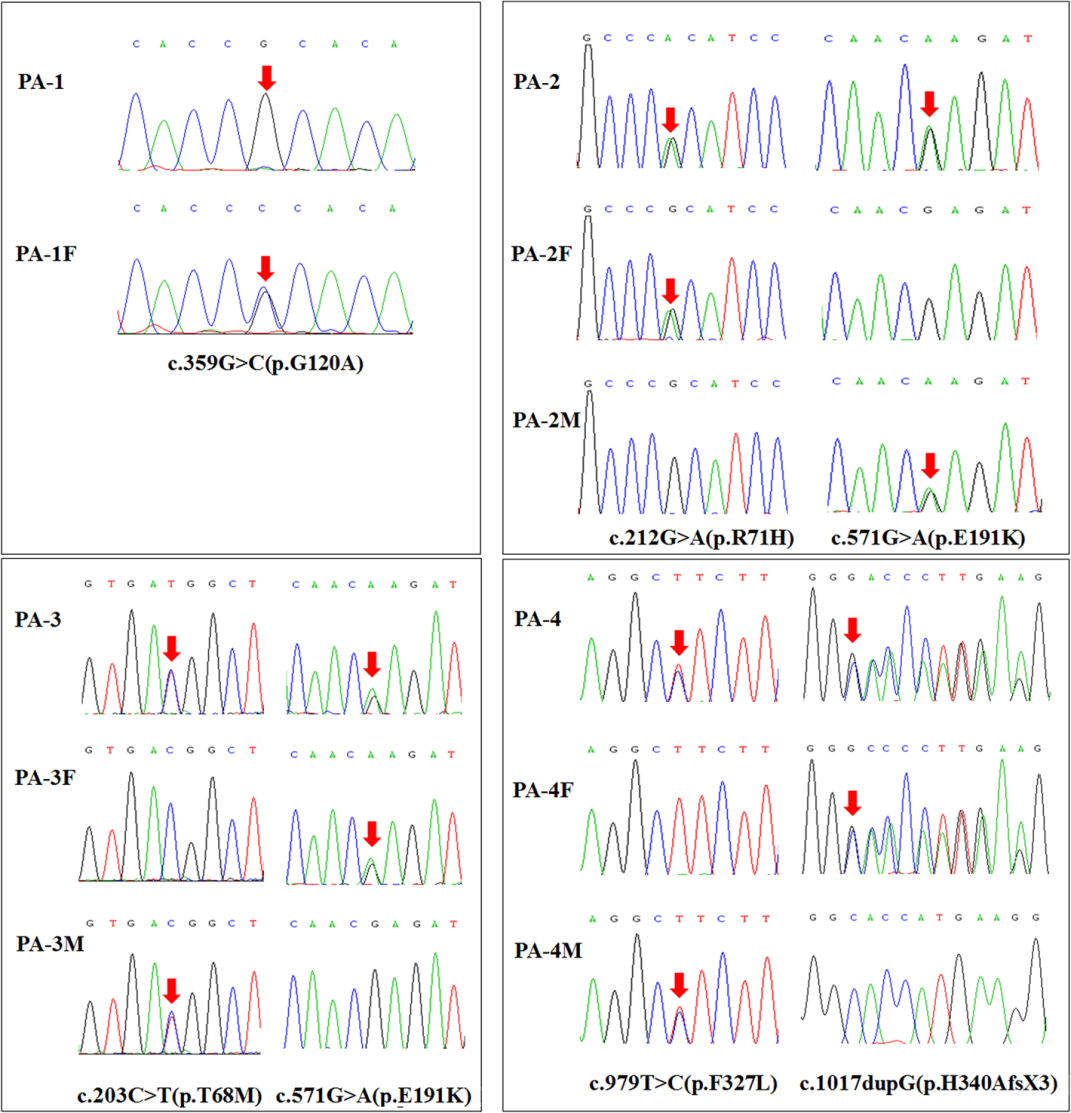
Chromatograms of *6 ALPL* mutations identified in the current study. Each frame represents the sequencing results of each pedigree

Six different variants were identified in the *ALPL* gene in our four patients, including five missense variants and one small insertion variant (Table 2). All variants were inherited from their unaffected parents except patient 1 (Fig. 2). As the most severe and lethal infantile HPP in the present study, patient 1 carried a novel homozygous variant of c.359G > C (p.G120A) at exon 5 of the *ALPL* gene, which was predicted to be disease-causing by in-silico analysis of bioinformatics software. His father demonstrated a heterozygous status, while his mother was not involved in this study. To further investigate the effect of the novel missense mutation of c.359G > C, a 3D structure modeling of TNSALP was used to locate the residue, indicating that the residue p.G120A was located in the secondary structure of the TNSALP homodimer interface.

**Table 2:** ALPL gene mutations in 4 HPP Chinese patients

Patient	Genotype	Exon	Nucleotide change	Amino acid change	Mutation type	Reported previously
PA-1	homozygous	5	c.359G>C	p.G120A	Missense	Novel
PA-2	Compound heterozygous	4	c.212G>A	p.R71H	Missense	Reported
		6	c.571G>A	p.E191K	Missense	Reported
PA-3	Compound heterozygous	4	c.203C>T	p.T68M	Missense	Reported
		6	c.571G>A	p.E191K	Missense	Reported
PA-4	Compound heterozygous	9	c.979T>C	p.F327L	Missense	Reported
		10	c.1017dupG	p.H340AfsX3	Frameshift	Novel

Patient 1 (PA-1) was homozygote, whose father (PA-1F) was heterozygous carrier, but his mother’s genotype was unknown (she was not detected). All of other 3 patients (PA-2, PA-3 and PA-4) were compound heterozygote whose variants were inherited from their parents. Arrows indicated the mutated sites. F means father and M means mother. 

### Literature review

In order to provide a comprehensive overview of Chinese patients with HPP, we reviewed all publications regarding Chinese HPP cases in the PubMed database (https://www.ncbi.nlm.nih.gov/pubmed) and the *ALPL* gene mutations database (http://www.sesep.uvsq.fr/03_hypo_mutations.php) [12–20].

**Fig. 3:**
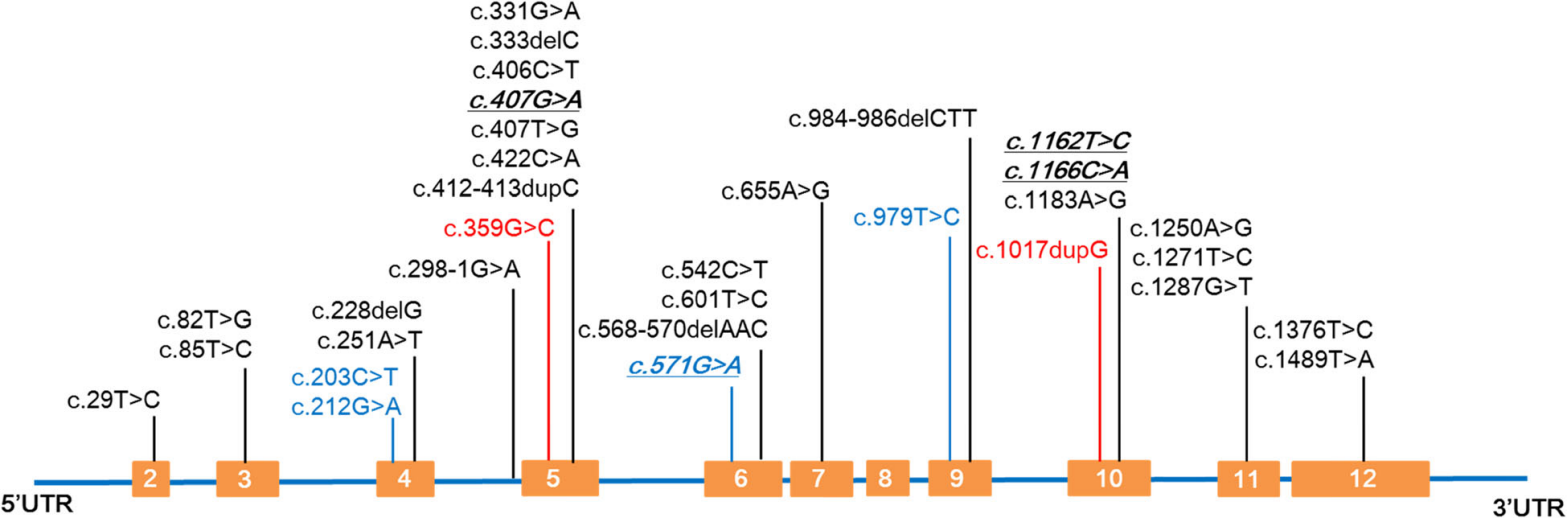
Schematic drawing of the *ALPL* gene mutations identified in Chinese HPP patients. The position of all identified mutations are indicated. The mutations reported previously are indicated in black, while six mutations identified in the present study are indicated in red and blue. The red color highlights the novel mutations, and the blue color represents the pathogenic mutations reported previously, whereas italic font with underline represents the mutations detected in two patients

The full spectrum of *ALPL* gene mutations in reported Chinese HPP patients is presented in Fig. 3. A total of 26 mutations from 15 Chinese families were previously reported, including 20 missense mutations, 4 small deletion mutations, one small insertion and one splice site mutation. The 26 mutations were distributed throughout all 12 exons of the *ALPL* gene except for exon 1 and 8, with mutations most often located at exon 5 (27.6%, 8/29). Three mutations (c.407G > A, c. 1162 T > C and c.1166C > A) were detected twice. No hot mutation was identified. The most common form of HPP in Chinese patients was childhood HPP, followed by adult HPP, whereas only one patient with onset of age at 1 month old was reported as infantile HPP. No lethal HPP has been reported in Chinese patients.

In the present study, three clinical forms of HPP were diagnosed in four Chinese children, which were infantile, childhood and odonto HPP. All six mutations (c.203C > T, c.212G > A, c.359G > C, c.571G > A, c.979 T > C and c.1017dupG) were first reported in the Chinese population. Of which, c.359G > C and c.1017dupG were novel mutations. Patient 1 carrying homozygous c.359G > C exhibited severe phenotype of lethal infantile HPP.

## Discussion and conclusions

The *ALPL* gene encoding TNSALP was located on chromosome 1p34–36. HPP is caused by loss of function mutations in the *ALPL* gene. The first case of HPP was reported in 1948 and the *ALPL* gene mutation was first reported in 1988 [1, 21]. HPP presents a heterogeneous phenotype ranging from life threatening to asymptomatic presentation. In general, the pattern of inheritance in HPP is autosomal recessive. However, HPP may also be transmitted by autosomal dominant and incomplete penetrance of dominant transmission [22]. The severe forms of HPP usually transmit by autosomal recessive, while milder forms of HPP more often present with autosomal dominant with variable expressivity. These heterogeneous clinical features and inheritance patterns result in the difficulty of timely diagnosis and challenging of genetic counselling [23, 24].

In the present study we investigated four Chinese children affected by three forms of HPP, which were lethal infantile, childhood and odonto HPP. All patients follow autosomal recessive inheritance pattern. They presented clinical symptoms early at 1 day old (patient 1) or at 1 year old (patient 2, 3 and 4). All four patients had low serum ALP activity. As reported, patients with perinatal or infantile HPP have high morbidity and mortality in the first 5 years of life [7]. Patient 1 was a typical infantile form of HPP who presented respiratory distress, nearly undetectable ALP activity and general skeletal hypomineralization. He died of respiratory failure at 3 months old, therefore was diagnosed as lethal infantile form of HPP. To the best we know, this was the first case of lethal infantile HPP reported in the Chinese population. Recent researches revealed that a rapidly worsening clinical course often occurs in prenatal [25] or infantile HPP mainly due to respiratory compromise [7]. However, further investigation is need to clarify the mechanism.

It has been reported that diagnostic delay is common due to limited awareness of HPP [26]. Patient 2 and 3 were Childhood HPP who presented premature loss of deciduous teeth and muscle weakness at 1 year old. Patient 3 also had joint swelling and bone pain starting at 1 year old, however, he was confirmed with HPP at 8 years old. Patient 4, although his early loss of deciduous teeth started at 1 year old, was diagnosed at 4 years old. These observation demonstrated that some HPP cases may not be recognized well and managed timely.

Molecular diagnosis provides great advantage to confirm the diagnosis of HPP. HPP is caused by a loss of function mutation in the *ALPL* gene encoding TNSALP. It has been reported that few mutations may be frequent in particular populations [23]. For instance, c.1559delT and p.F327 L are two common mutations in the Japanese population, whereas c.571G > A (p.E191 K) is identified in half of European patients with moderate HPP [27, 28]. To elucidate the mutational characteristics of the *ALPL* gene in the Chinese population, we reviewed all reported Chinese HPP cases in the literature. We found that the most mutations in Chinese HPP patients were missense variants located in exon 5 in the *ALPL* gene. No frequent mutations were recognized (Fig. 3). In the present study, six *ALPL* mutations, including five missense mutations and one splicing mutation, were identified. All of these mutations were first reported in the Chinese population.

It is considered that the variety of *ALPL* mutations results in highly variable clinical expressivity, resulting in difficulty to assess the severity of a novel mutation in the *ALPL* gene [15]. The severity of HPP symptoms was correlated with the level of ALP activity affected by the *ALPL* mutation [29]. Severe HPP often exhibit very low level of ALP activity, whereas mild HPP usually retain most ALP activity. Severe form manifests early, whereas mild form may be diagnosed in adulthood [30]. Patient 1 was a homozygote of c.359G > C which has not been previously described in the literature. However, typical clinical features, very low level of serum ALP activity, overall hypomineralization and homozygote status all were consistent with severe infantile form of HPP, indicating the variant c.359G > C seems to be at a crucial position of ALP protein. Further investigation revealed that the variant c.359G > C (p.G120A) is located in the homodimer interface, a crucial site of secondary structure in the TNSALP and highly conserved throughout many species. Thus, the novel mutation c.359G > C was strongly indicated to be disease-causing and related to severe infantile form of HPP. Further functional study of the mutation c.359G > C is needed.

Patient 2, 3 and 4 were compound heterozygote in the *ALPL* gene. Patient 2, the childhood HPP, had been identified with two known pathogenic variants, c.212G > A (p.R71H) at exon 4 and c.571G > A (p.E191K) at exon 6 [31, 32]. As another childhood HPP, Patient 3 carried the identical c.571G > A (p.E191K) variant of Patient 2 and another pathogenic c.203C > T (p.T68 M) variant at exon 4 which was also reported previously [33]. Interestingly, the variant c.571G > A (p.E191 K) in Patient 2 and 3, was the common mutation reported in European population with moderate HPP. Patient 4 was the mildest odonto HPP in the present study. He demonstrated c.979 T > C (p.G120A) and c.1017dupG (p. H340AfsX3). The c.979 T > C (p.G120A) variant was pathogenic reported previously [34], whereas the novel variant c.1017dupG (p.H340AfsX3) was predicted to result in a translation frameshift and premature protein termination (p. H340AfsX3).

Some limitations exist in the present study. First, the number of HPP patients is not big enough to reveal the phenotype–genotype correlations. Second, PLP, the best markers of HPP, was not detected in the present study since the method is still not available in our center. Future study will be focused on collecting more data to reveal the correlation between phenotype and genotype of HPP in the Chinese population.

Currently enzyme replacement therapy with asfotase alfa (Strensiq™, USA) is currently the only approved treatment for HPP. Asfotase alfa can restore normal ALP levels and prevented skeletal and dental manifestations of HPP. Unfortunately it has been approved in many countries but not in China. None of the patients in the present study have received asfotase alfa for treatment.

In the present study, we described the clinical and genetic characteristics of HPP in four unrelated Chinese pedigrees who were affected with infantile, childhood and odonto forms of HPP. All patients followed autosomal recessive inheritance pattern. Six mutations in the ALPL gene were identified including four known mutations and two novel mutations. The novel missense mutation (c.359G > C) caused the decrease of ALP activity and was related to lethal infantile form of HPP. The study expanded knowledge about the characteristics of HPP.

## Data Availability

Not applicable. We are submitting the data together with this manuscript.

## Abbreviations

ALP: Alkaline phosphatase
HPP: Hypophosphatasia
PEA: Phosphoethanolamine
PLP: Pyridoxal-5-phosphate
PPi: Inorganic pyrophosphate
TNSALP: Tissue Non-specific Alkaline Phosphatase
